# A Systematic Review of the Use of St. John’s Wort for Smoking Cessation in Adults

**DOI:** 10.7759/cureus.18769

**Published:** 2021-10-14

**Authors:** Namrata Walia, Sandra Gonzalez, Roger Zoorob

**Affiliations:** 1 Family and Community Medicine, Baylor College of Medicine, Houston, USA

**Keywords:** systematic review, cigarette smoking, tobacco cessation, complementary alternative medicine, st. john’s wort

## Abstract

St. John’s wort (SJW) has been researched and clinically used for treating various psychiatric disorders, including depression. Few clinical trials have studied its use in smoking cessation. This systematic review provides comprehensive evidence of the studies conducted to date. Five databases were searched for randomized controlled trials (RCTs) that evaluated the effectiveness of SJW for smoking cessation in adults. The trials included the use of SJW alone, or along with nicotine replacement therapy, chromium, or behavioral therapies. The Preferred Reporting Items for Systematic Reviews and Meta-Analyses guidelines were used to report this systematic review. Overall, four RCTs met the eligibility criteria, and the risk of bias analysis was conducted using the Cochrane criteria. Abstinence, along with other physical symptoms, were measured as outcomes at the end of the follow-up period. Studies reported variable abstinence rates and a decrease in cravings at the end of 12-14 weeks. However, there was minimal to no difference reported between the intervention and placebo groups in all of the clinical trials. One of the studies reported minimal physical side effects. Overall, SJW was well tolerated. Quality analysis of the included studies showed low concerns in two studies while the other two studies showed high concerns in the risk of bias judgment. More clinical trials with larger sample sizes should be conducted in the future to evaluate the effectiveness of the use of SJW for smoking cessation.

## Introduction and background

Cigarette smoking is the leading cause of preventable disease and death in the United States [1]. Although the prevalence of adult smokers has declined in the past few decades, the estimate of current adult smokers increased from 13.7% adults in 2018 to 20.8% in 2019 [1,2]. Despite the known ill effects on health, a variety of combustible and non-combustible tobacco products are available in the United States. In 2019, around 50.6 million adults (20.8% of the US population) reported current use of any tobacco product. The majority of users reported using cigarettes, followed by e-cigarettes and cigars [1]. The Centers for Disease Control and Prevention (CDC) reported in 2015 that approximately two-thirds of cigarette smokers were interested in quitting smoking [3]. However, less than one in ten smokers were successful [3]. Evidence-based smoking cessation programs, including pharmacological and behavioral interventions, have successfully helped people to quit smoking [4-9]. These programs have shown to be cost-effective treatment options in clinical and community settings [10-14].

Complementary and alternative medicine has been shown to be a cost-effective treatment modality compared to conventional therapy alone in many medical conditions [15]. *Hypericum perforatum*, popularly called St. John’s wort (SJW), was originally identified as an herb by Greek and Roman physicians [16]. In modern times, it has been extensively used in western countries such as the United States, the United Kingdom, and Germany. The herb has been reported to have antibacterial, antiviral, and anti-inflammatory properties [17]. It has been extensively studied in clinical trials as a treatment for pain [17], autism [18], major depressive disorder [19,20], and obsessive-compulsive disorder [21]. Recently, SJW has been studied as a treatment for smoking cessation. This systematic review provides comprehensive evidence from the literature and analyzes the consolidated results.

## Review

Methodology

This review included randomized controlled trials (RCTs) that studied the effectiveness of SJW as a treatment modality for smoking cessation in adults aged 18 years and over. There was no limitation on the independent or combined use of SJW with other pharmacologic treatments. Non-randomized, quasi-experimental, and observational studies were excluded. Multiple databases, namely, Medline, Embase, Cochrane, Scopus, and PsychINFO, were used for a systematic search of published studies. The search methodology used keywords such as “St. John’s wort,” “*Hypericum*,” “smoking cessation,” “tobacco,” and MeSH terms such as “Tobacco-related.” Gray literature was searched using the Google search engine and no additional relevant articles were found.

The data extraction and screening were conducted by a single reviewer. In case of uncertainty, the other two co-authors were consulted for a consensus-based discussion. The data were extracted using a predesigned data extraction form. The form included data on article name and author(s), journal information and publication date, sample size, eligibility criteria for studies, details on treatment and intervention groups, and results of the trials conducted. The quality of the trials was assessed using Cochrane Risk of Bias tool version 2 (ROB2) [22]. This tool assesses the trials based on five domains, namely, bias due to randomization process, deviations from intended interventions, missing outcome data, measurement of outcome, and selection of reported result. The overall score was reported as H (high risk of bias), S (some concerns), and L (low risk of bias).

Results

**Figure 1:**
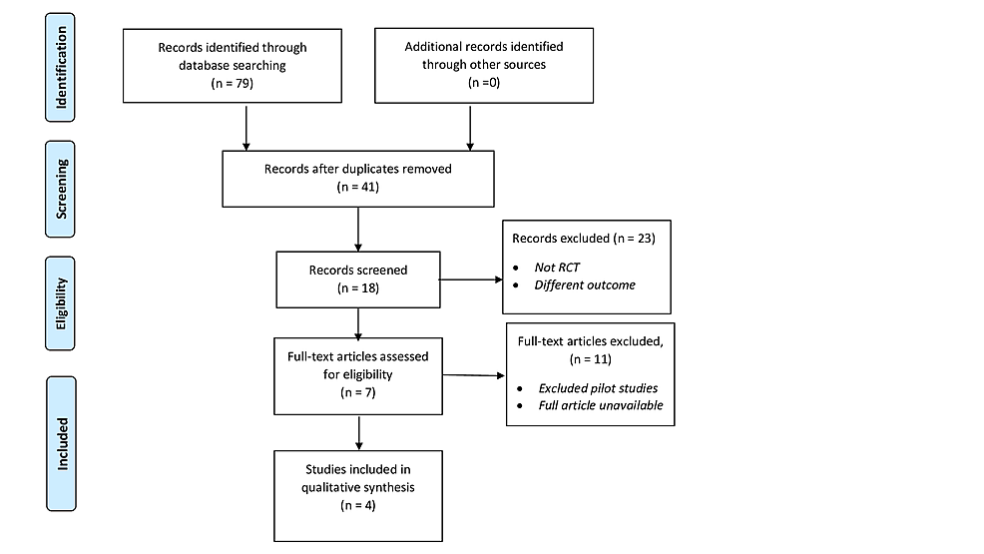
PRISMA flow diagram. PRISMA: Preferred Reporting Items for Systematic Reviews and Meta-Analyses

A total of 79 articles were found across all the databases searched. After removing duplicates and screening the abstracts, four studies met the eligibility criteria. The Preferred Reporting Items for Systematic Reviews and Meta-Analyses (PRISMA) guidelines were used for the reporting of this systematic review. Figure 1 shows the flow of the process in selecting the eligible studies.

Demographics and Sample Characteristics

Out of the four eligible studies, two were conducted in the United States, and the other two were conducted in the United Kingdom and Australia. Table 1 shows that the sample size for the studies ranged from 20 to 143 participants. Both male and female participants, over the age of 18, were included in the RCTs. Participants had a smoking history of 10 or more cigarettes per day; one of the studies required an additional five or more years of smoking and a prior quitting attempt as an eligibility criterion.

**Table 1:** Study characteristics and outcomes of eligible studies. SJW: St. John’s Wort; NRT: nicotine replacement therapy; CES-D: Center for Epidemiologic Studies Depression; MNWS: Minnesota Tobacco Withdrawal Scale; DAST-20: Drug Abuse Screen Test 20; CAGE: Cut down, Annoyed, Guilty, and Eye-opener; MPSS: Mood and Physical Symptoms Scale; OR: odds ratio; CI: confidence interval; SE: standard error

Source	Country	Sample size	Age	Smoking history	Intervention	Duration	Outcome	Outcome measures	Result
Camfield et al. (2012) [23]	Australia	20	18–60 years	>5 years. Currently smoking >10 cigarettes/day; at least one prior attempt at quitting	SJW, NRT, or both	14 weeks; Baseline: 4 weeks prior to quitting	Neurocognitive reaction time and accuracy	Spatial working memory task; steady-state probe topography; smokerlyser to confirm abstinence	The mean reduction in the number of cigarettes smoked per week at week 14, in comparison to baseline, was 55.93 cigarettes for the SJW group, 61.80 cigarettes for the NRT group, and 49.15 cigarettes for the SJW and NRT combined group. The treatment groups showed similar efficacy in improving cognitive effects
Sood et al. (2010) [24]	United States	118	Over 18 years	10 cigarettes per day in the past year	300 mg, 600 mg, or placebo three times a day plus behavioral intervention	12 weeks (additional 12 weeks follow-up)	Abstinence and craving	Fagerstrom test for nicotine dependence; CES-D scale; contemplation ladder to measure readiness to quit; MNWS with smoking diary; Beck Depression Inventory, second edition, CAGE, and DAST-20	The mean cigarettes per day did not differ significantly between groups 8.77.2 (n = 15) placebo; 9.59.3 (n = 12) for the 300 mg group; and 9.56.4 (n = 16) for the 600 mg group. Nicotine withdrawal and craving were both observed to decrease significantly with time (withdrawal:estimate = −0.019 per day, SE = 0.008, p = 0.016; craving:estimate = −0.031 per day, SE = 0.01, p = 0.018). However, no significant differences were observed in abstinence rates at 12 and 24 weeks between SJW dose groups and placebo
Parsons et al. (2009) [25]	United Kingdom	143	Over 18 years	>10 cigarettes per day	900 mg SJW and 400 m chromium; and placebo	14 weeks; treatment started 2 weeks before	Abstinence and change in weight	MPSS-C score (urge to smoke), MPSS-M score (mood changes), MPSS-P score (physical symptoms)	Participants on active SJW (8.5%) and placebo (12.5%) achieved prolonged abstinence at 4 weeks, with an OR (95% CI) of 0.65 (0.22–1.92). At 6 months, SJW active (4.2%) and SJW placebo (8.3%) participants were still abstinent, with an OR of 0.49 (0.12–2.02). The mean difference in weight gain between active chromium and placebo was −0.81 kg (−3.79 to 2.18) at 4 weeks and −3.88 kg (−12.13 to 4.38) at 6 months
Lawvere et al. (2006) [26]	United States	37	18–65 years	10 cigarettes/day	SJW 450 mg plus cessation counseling messages	12 weeks	Abstinence, weight change, and physical changes	Carbon monoxide testing; Shiffman-Jarvik for withdrawal symptom scale; anxiety and depression scale	A quit rate of 37.5% (95% CI: 21.1%, 57.5%) with nine of the original 37 subjects who started taking SJW smoke-free at 12 weeks. No significant change in weight was noted. Use of SJW was generally well tolerated

Intervention Details

Table 1 describes the intervention and placebo of the selected studies. The studies investigated the effectiveness of SJW alone or in combination with nicotine replacement therapy (NRT), chromium, behavioral therapy, and cessation counseling messages. The studies followed their participants for 12-14 weeks until the primary data collection timepoint. One of the studies by Sood et al. [23] followed the participants for an additional 12 weeks for collecting secondary data. Table 1 lists all the varied outcome measures used.

Outcomes

Camfield et al. [24] conducted an RCT to evaluate neurocognitive effects (memory function test for reaction time and accuracy) during the use of SJW for smoking cessation. The authors used spatial working memory task, steady-state probe topography, and smokerlyser to confirm abstinence from smoking. The mean reduction in the number of cigarettes smoked per week at week 14, in comparison to baseline, was 55.93 cigarettes for the SJW group, 61.80 cigarettes for the NRT group, and 49.15 cigarettes for the SJW and NRT combined group. The interaction between treatment groups (SJW, NRT, or NRT + SJW) and study visit (baseline vs. retest) (F (2,17) = 1.674; p > 0.05) and between treatment groups and time (F (2, 17) = 0.415; p > 0.05) were found to be non-significant. However, pairwise comparisons revealed that, at retest, the reaction time was faster for the SJW group compared to the NRT group (p = 0.116). The authors interpreted that lack of results (or lack of difference in groups) could mean similar efficacy of the treatment groups.

Sood et al. [23] examined the efficacy of SJW for smoking cessation using two different oral doses (300 or 600 mg). The authors used the Fagerstrom test for nicotine dependence, the Center for Epidemiologic Studies Depression Scale (CES-D), the contemplation ladder to measure readiness to quit, the Minnesota Tobacco Withdrawal Scale (MNWS) with smoking diary, the Beck Depression Inventory, Cut down, Annoyed, Guilty, and Eye-opener (CAGE), and Drug Abuse Screen Test 20 (DAST-20). The mean cigarettes smoked per day did not differ significantly between groups 8.77.2 (n = 15) and placebo; 9.59.3 (n = 12) for the 300 mg group and 9.56.4 (n = 16) for the 600 mg group. Nicotine withdrawal and craving were both observed to decrease significantly with time (withdrawal:estimate = −0.019 per day, standard error [SE] = 0.008, p = 0.016; craving:estimate = −0.031 per day, SE = 0.01, p = 0.018). Using the intention-to-treat analysis, no significant differences were observed in abstinence rates at 12 and 24 weeks between SJW dose groups and placebo. SJW did not attenuate withdrawal symptoms among abstinent subjects. However, no significant side-effects were noted with SJW.

Parsons et al. [25] examined the combined use of SJW and chromium to aid in smoking cessation and prevent associated weight gain. The authors used the Mood and Physical Symptoms Scale (MPSS)-C score to measure urge to smoke, MPSS-M score for mood changes, and MPSS-P score to measure physical symptoms such as constipation, mouth sores, and sore throat. Participants on active SJW (8.5%) and on placebo (12.5%) achieved prolonged abstinence at four weeks, with an odds ratio (OR) (95% confidence interval [CI]) of 0.65 (0.22-1.92). At six months, SJW active (4.2%) and SJW placebo (8.3%) participants were still abstinent, with an OR of 0.49 (0.12-2.02). The mean difference in weight gain between active chromium and placebo was −0.81 kg (−3.79 to 2.18) at four weeks and −3.88 kg (−12.13 to 4.38) at six months. There were no significant differences in urges to smoke or tobacco withdrawal symptoms. However, it was safe and well tolerated.

Lawvere et al. [26] examined the feasibility and efficacy of SJW for smoking cessation. The authors used carbon monoxide (CO) testing, Shiffman-Jarvik for withdrawal symptom scale, and anxiety and depression scale. The 12-week quit rate was nine participants or 37.5% (95% CI: 21.1%, 57.5%); 13 (54%) participants were smoke-free at the three-week visit. No significant change in weight was noted. Participants reported physical symptoms such as a change in bowel movements (8.8%), sensitivity to light (5.9%), constipation (5.9%), abdominal pain (2.9%), dizziness (2.9%), and fatigue (2.9%). Overall, the use of SJW was well tolerated.

Risk of Bias Assessment

Cochrane Risk of Bias tool for randomized trials [22] was used to assess the risk of bias and quality of trials. Table 2 shows that two out of the four eligible studies showed low concerns, while the other two studies showed high concerns regarding the risk of bias judgment. Two studies did not have information if the outcome assessors were blinded from the randomization. This led to high concerns regarding the risk of bias. The studies also did not address or change the statistical analysis plan due to missing data.

**Table 2:** Risk of bias judgment (low, some concerns, high).

	Studies			
	Camfield et al. [23]	Sood et al. [24]	Parsons et al. [25]	Lawvere et al. [26]
Risk of bias arising from randomization process	Low	Low	Low	Low
Risk of bias due to intended intervention (effect of assignment to intervention)	Low	Low	Low	Low
Risk of bias due to intended intervention (effect of adhering to intervention)	Low	Low	Low	Low
Risk of bias due to missing outcome data	Some concerns	Low	Low	Low
Risk of bias in measurement of the outcome	High	Low	High	Low
Risk of bias in selection of the reported result	Low	Low	Some concerns	Low
Overall risk of bias	High	Low	High	Low

Discussion

To our knowledge, this is the first systematic review to analyze the efficacy of the use of SJW for smoking cessation. The studies included in this review used a combination of SJW with NRT, behavioral interventions, or chromium to curbing weight gain caused by smoking cessation. The authors showed a reduction in the number of mean cigarettes, but no significant difference was reported between the different groups or placebo. The groups showed similar effects on their respective outcomes, including cognitive effects, changes in weight or physical symptoms, and craving. The results of this systematic review of four RCTs, with a total of 318 participants, did not provide sufficient evidence for using SJW for smoking cessation. However, further randomized trials with larger sample sizes should be conducted in the future.

The safety profile of complementary and alternative medicine has been well studied and reported. Most types of complementary and alternative medicine are safe to use, with minimal to no side effects. SJW, in particular, has been shown to be safe and effective in patients experiencing symptoms of depression. In the clinical studies included in this review, SJW was well tolerated, and no significant side effects were experienced by patients. However, the participants using SJW in the studies reported minor physical side effects including headaches, nausea and vomiting, diarrhea, fatigue, dizziness, dry mouth, and insomnia at least once during the study period.

Smoking cessation attempts have been associated with symptoms such as anxiety, depression, and mood changes [27]. The clinical efficacy of the use of SJW for psychiatric disorders such as major depressive disorders has been previously reported in the literature [19,20]. Because smoking and depression have common neurobiological mechanisms, the researchers and clinicians assumed a similar effect of SJW on smoking cessation. However, the RCTs conducted to date did not show any difference in the use of SJW and placebo for aiding smoking cessation.

Participant treatment adherence and drop-outs are an issue for interventional clinical studies for addictive substances. The social and biological phenomenon behind the addiction of smoking and the chronic use of tobacco induces structural and functional changes in the brain. The re-wiring or re-educating the brain during smoking cessation attempts requires psychosocial support in addition to pharmacotherapy. The lack of such interventions in a clinical study determining the efficacy of a treatment modality could provide inconclusive results. An integrative approach including interventions targeting the psychosocial factors is recommended to test the treatment in a real-life environment.

Study limitations

A meta-analysis could not be conducted due to the high risk of bias in most studies. Moreover, the studies used different statistical parameters to report their results, due to which a meta-analysis was not feasible. Lastly, RCTs are not always the most suitable study design. Future systematic reviews should be conducted including cross-sectional studies to evaluate the use and efficacy of SJW as a smoking cessation aid.

## Conclusions

This review demonstrated the potential of SJW as an adjunct treatment modality for smoking cessation. However, the small number of RCTs reported in the literature, and included in the systematic review, failed to provide evidence in favor of the effectiveness of SJW for smoking cessation. Further clinical trials with larger sample sizes, possibly conducted with a different study design, or high-quality trials of intervention should be conducted in the future to evaluate the effectiveness of the use of SJW for smoking cessation.
